# ﻿First record of silk-loving genus *Embiophila* (Hemiptera, Heteroptera, Plokiophilidae) from Asia, with description of a new species from China

**DOI:** 10.3897/zookeys.1248.155672

**Published:** 2025-08-08

**Authors:** Haoyang Xiong, Zhuo Chen, Hu Li, Wanzhi Cai

**Affiliations:** 1 State Key Laboratory of Agricultural and Forestry Biosecurity, Department of Entomology, College of Plant Protection, China Agricultural University, Beijing 100193, China China Agricultural University Beijing China

**Keywords:** Embiophilini, Embioptera, new species, Oriental Region, taxonomy

## Abstract

The silk-loving bug *Embiophilasinica***sp. nov.** is described from Yunnan, southwestern China, representing the first record of the genus *Embiophila* (Heteroptera, Plokiophilidae) from Asia. The new species can be distinguished from its congeners by the different number of the spines on the fore – and mid-femora, and the numbers of the corial glands on the hemelytra. Photographs of the habitus and diagnostic characters, as well as living individuals in natural habitats, are provided. A key to *Embiophila* species is presented. The ecology of the new species and the distribution of *Embiophila* are briefly discussed.

## ﻿Introduction

The Plokiophilidae comprises a small group of minute and distinctively behavioral bugs, with nine genera and 22 species known worldwide (Štys 1967; Henry 2020; Luo et al. 2021). Most of these bugs inhabit spider webs, while a minority of species have been observed living on the webs of embiopterans, one species is considered to be free living, and the living habits of the other species remain unknown (Štys and Baňař 2016; Schuh and Weirauch 2020; Luo et al. 2021).

The first species of Plokiophilidae, *Arachnophilacubana* China & Myers, 1929, was described as a member of the Microphysidae (China and Myers 1929). Due to the homonymy with *Arachnophila* Salvadori, 1874 (Salvadori 1874), China (1953) renamed *Arachnophila* China & Myers, 1929 as *Plokiophila* China, 1953, and described the second species, *Embiophilamyersi* China, 1953, which inhabited the webs of the webspinners (the order Embioptera). This second species exhibits significant morphological differences from *Plokiophila*, which lead him to establish a new genus, *Embiophila* China, 1953, to accommodate it, while also elevating Plokiophilinae to the subfamilial rank within the Microphysidae. Carayon (1961) elevated Plokiophilinae as a distinct family and proposed the subfamilies Plokiophilinae and Embiophilinae. Carayon (1974) published the first monograph on Plokiophilidae, in which he described one new genus and seven new species, comprehensively elaborated on the morphology and habits of the family, and summarized previous knowledge. More species of the Plokiophilidae were discovered in the subsequent decades (Schuh and Štys 1991; Eberhard et al. 1993; Carpintero and Dellapé 2005; Schuh 2006; Popov 2008; Schuh et al. 2008). Schuh et al. (2015) downgraded the Embiophilinae to a tribe of the Plokiophilinae based on male and female genital structures, proposed a new subfamily, Heissophilinae, and a new tribe, Lipokophilini, and described two new genera and three new species. Subsequent new genera and new species of the Plokiophilidae were successively described since then (Štys and Baňař 2016; Luo et al. 2021), while the classification of the family has not been revised again.

The genus *Embiophila* is a unique group of the Plokiophilidae, whose species are generally living on the webs of embiopterans but also have been found to be free-living (Carayon 1974). China (1953) described *Embiophilamyersi* based on specimens collected from Trinidad and Tobago and established *Embiophila* to accommodate this species. Carayon (1974) further divided *Embiophila* into the subgenera *Acladina* and *Embiophila* s. str. based on the venation of the hind wings, the length of the labial segments, and the morphology of the mid tibia, and described the second species of the genus, E. (A.) africana Carayon, 1974 from Congo. At the same time, he suggested that the genus Embiophila of the Old World might belong to the subgenus Acladina, while those of the New World belong to the subgenus Embiophila. The third species, E. (E.) maesi Carpintero & Dellapé, 2005, was discovered in Nicaragua (Carpintero and Dellapé 2005).

During the recent field investigations in Yunnan, southwestern China, we collected several specimens of a plokiophilid species on the webs of an unidentified embiopteran. Further examinations revealed that it represents a new species of *Embiophila*, and the description of the species constitutes the subject of this study. This new species represents the first record of *Embiophila* in China as well as Asia. The identification key to *Embiophila* species is updated, and the ecology of the new species and the distribution of the genus are briefly discussed.

## ﻿Materials and methods

Specimens examined in this study were deposited in the Entomological Museum of China Agricultural University, Beijing, China (**CAU**).

External and genital structures were examined using a Nikon SMZ745 stereoscopic microscope. Measurements (in mm) were taken using a Nikon SMZ18 with the Adobe Photoshop 2023. Male genitalia and female abdomen were macerated in 10% potassium hydroxide solution (KOH) at 60 °C for 3 h. Photographs were taken using a Canon EOS 7D Mark II camera, with the habitus pictures taken using the Nikon SMZ18, wings and female abdomen photographs taken using Olympus 4×/0.10, and photographs of the legs were taken with Olympus 10×/0.25, male genitalia photographs with Olympus 20×/0.40. Ecological photos were taken with a Nikon D5 camera with a Laowa 100 mm f/2.8 lens. Scanning electron micrographs were prepared using a Regulus 8100 Scanning Electron Microscope at State Key Laboratory for Biology of Plant Diseases and Insect Pests, Institute of Plant Protection, Chinese Academy of Agricultural Sciences, Beijing, China. Figure plates were prepared using Adobe Photoshop 2023. Distribution map was prepared using QGIS Desktop v. 3.34.11.

The classification system of Plokiophilidae follows Schuh et al. (2015). Morphological terminology mainly follows Carayon (1974) and Schuh et al. (2015). In terms of measurements, length of body is defined as the straight-line distance from the anterior tip of the head to the posterior end of the abdomen; maximum width of pronotum is defined as the distance between the two posterolateral angles of the pronotum; greatest length of pronotum is defined as the perpendicular distance from the anterior margin of the pronotum to the line connecting the two posterolateral angles.

Abbreviations used in the text and figures are as follows:

**a** acus;

**ap** articulatory apparatus;

**cg** corial gland;

**Cgs** corial glands;

**Cu** cubitus;

**e** egg;

**lp** left paramere;

**M** media;

**R** radius;

**rp** right paramere;

**Sc** subcostal;

**sv** secondary vein;

**ts1** first segment of tarsus;

**ts2** second segment of tarsus;

**1An** first anal vein.

## ﻿Taxonomy

### ﻿Genus Embiophila

Taxon classificationAnimaliaHemipteraPlokiophilidae

China, 1953

CFBDE71D-AF42-5B45-9862-DCD69BD78687

Embiophila China, 1953: 67. Type species by original designation: Embiophilamyersi China, 1953.

#### Diagnosis.

*Embiophila* can be distinguished from other genera of Plokiophilidae by the following combination of characters: head lacking elongate neck behind eyes; legs relatively short and stout; fore and mid femora armed ventrally with spines; tarsi two-segmented, relatively short; hemelytron with distinct cuneus; pygophore tubular.

#### Diversity and distribution.

The genus currently contains four species, one from the Afrotropical Region (Carayon 1974), two from the Neotropical Region (China 1953; Carpintero and Dellapé 2005), and one described herein from the Oriental Region (Fig. 5).

### ﻿ Embiophila sinica sp. nov.

Taxon classificationAnimaliaHemipteraPlokiophilidae

AE716788-662A-5523-B302-9AB582CB91E2

https://zoobank.org/AAB7BB06-1798-4FA7-AC01-A944976BCAA7

**Figure 1.**
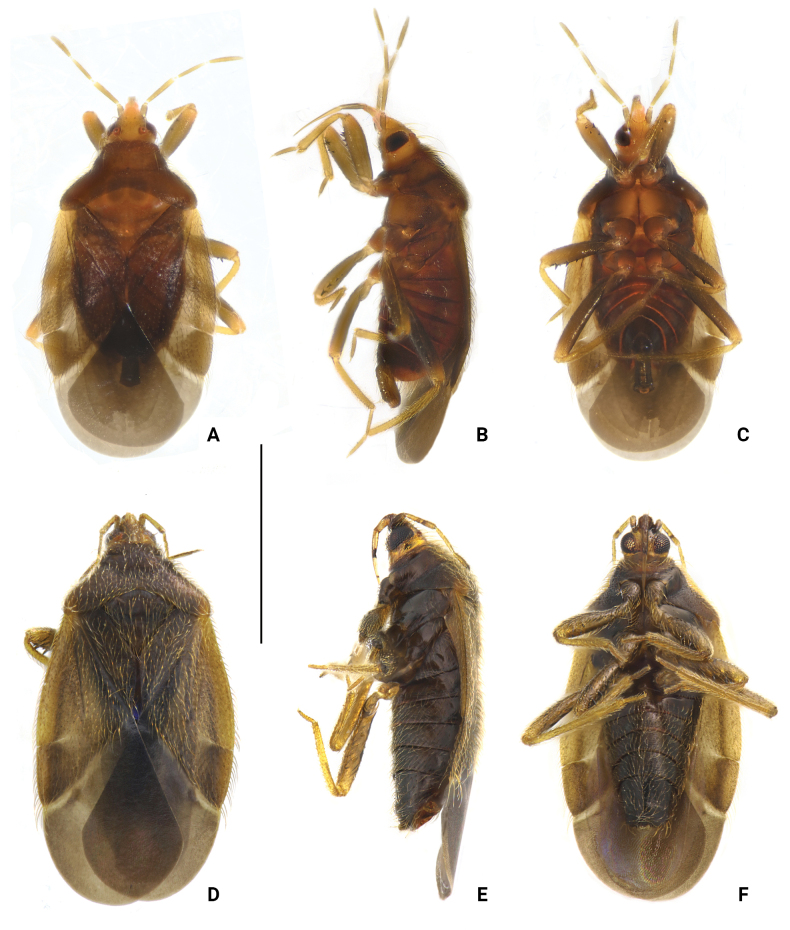
*Embiophilasinica* sp. nov., habitus. A. Male, holotype, dorsal view; B. Same, lateral view; C. Same, ventral view; D. Female, paratype, dorsal view; E. Same, lateral view; F. Same, ventral view. Scale bar: 1.5 mm.

**Figure 2.**
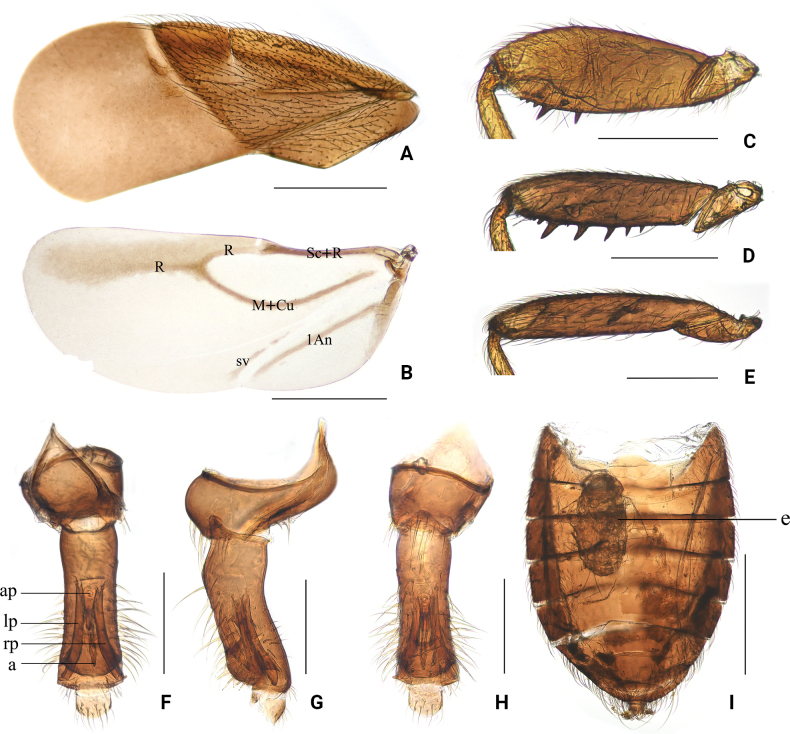
*Embiophilasinica* sp. nov., diagnostic morphological characters. A. Hemelytron, dorsal view; B. Hind wing, dorsal view; C. Fore femur, lateral view; D. Mid femur, lateral view; E. Hind femur, lateral view; F. Male genitalia, dorsal view; G. Male genitalia, lateral view; H. Male genitalia, ventral view; I. Female abdomen, dorsal view. Abbreviations: a = acus; ap = articulatory apparatus; e = egg; lp = left paramere; rp = right paramere. Scale bar: 0.5 mm (A, B, I); 0.25 mm (C–E); 0.2 mm (F–H).

**Figure 3.**
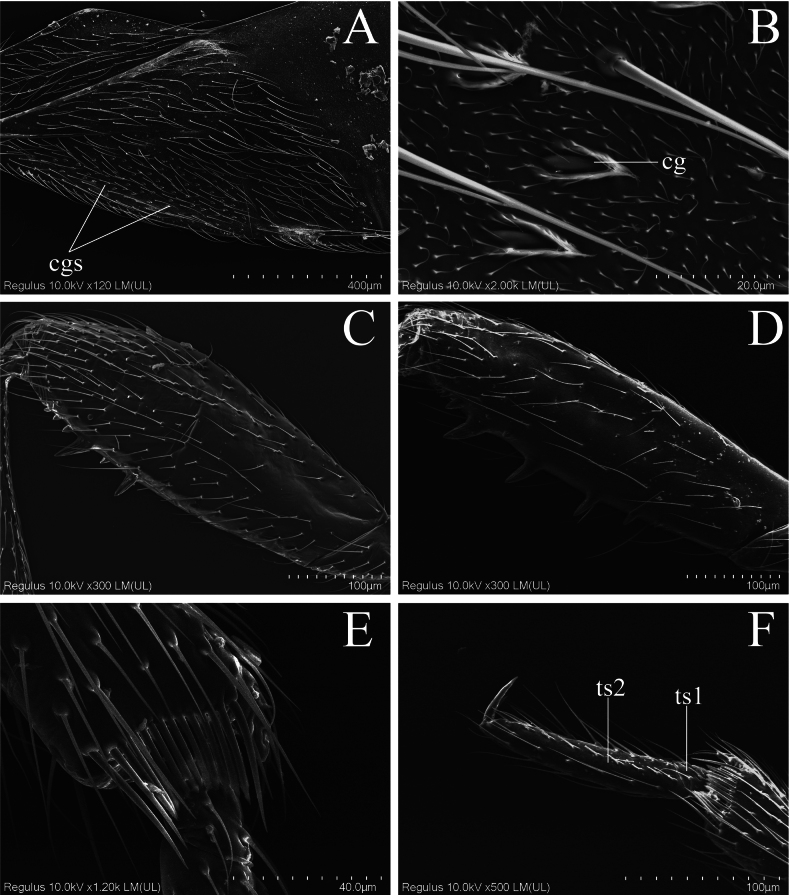
Scanning electron micrographs of *Embiophilasinica* sp. nov. A. Hemelytron, dorsal view (with all corial glands) B. Detail of corial gland, dorsal view; C. Fore femora, lateral view; D. Mid femora, lateral view; E. Apex of fore tibia, lateral view; F. Fore tarsus, lateral view. Abbreviations: cg = corial gland; cgs = corial glands; ts1 = first segment of tarsus; ts2 = second segment of tarsus.

**Figure 4.**
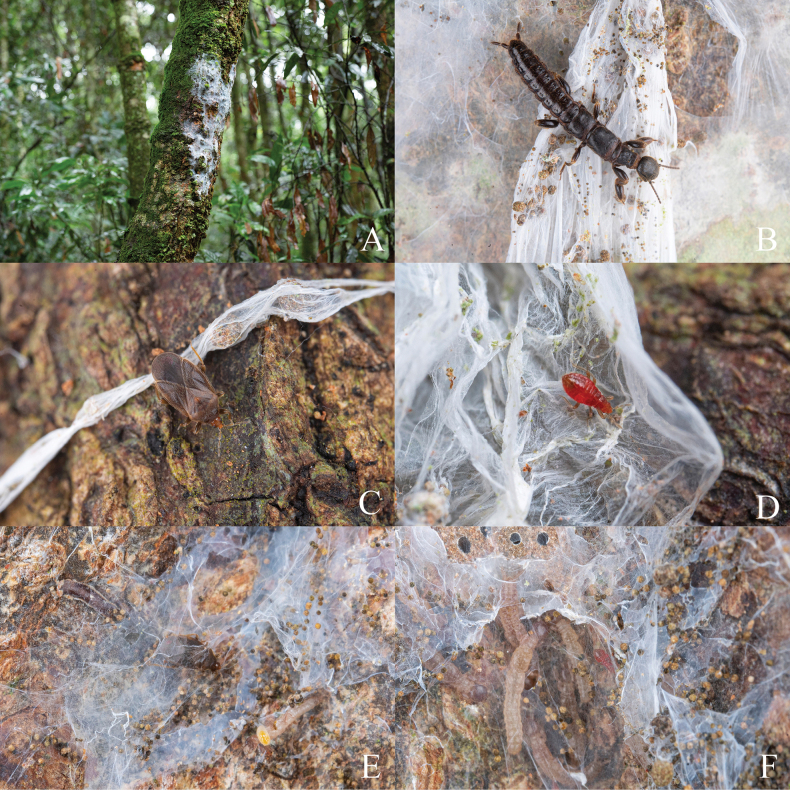
Habitats and living individuals of *Embiophilasinica* sp. nov. with embiopteran host. A. Habitat in type locality; B. Embiopteran host; C. Adult female of *Embiophilasinica* sp. nov. D. Nymph of *Embiophilasinica* sp. nov. E. Adult female of *Embiophilasinica* sp. nov. inside in web; F. Nymph of *Embiophilasinica* sp. nov. inside in web with embiopterans.

Figs 1 , 2 , 3 , 4 Chinese common name: 中华喜丝蝽

#### Type material.

***Holotype***: • ♂, China, Yunnan, Pu’er, Simao, Taiyanghe National Forest Park, 22.6179°N, 101.0902°E, ca. 1580 m, 12.ix.2024, leg. Haoyang Xiong, Zhuo Chen & Ruiyang Xian (CAU, accession number: CAUYN-PLO2). ***Paratypes***: • 2♂♂, 9♀♀ and 5 nymphs, same data as for holotype (CAU, accession number: CAUYN-PLO2).

#### Diagnosis.

This species is recognized within the genus by the following combination of characters: body length 1.9–2.2 mm, generally dark reddish brown; fore femora armed ventrally with 10 spines arranged in two rows (each with five) (Fig. 3C); mid femora armed ventrally with 13 spines arranged in two rows, outer with 11 and inner with two (Fig. 3D); exocorium with ca. 60 corial glands (Fig. 3A).

#### Description.

Macropterous male and female (Fig. 1). **Colouration.** Generally dark reddish brown. Tylus fulvous; eyes blackish brown; ocelli red. Antennal segment I dark yellowish brown; segments II–IV light brown. Labium yellowish brown. Scutellum and mesosternum reddish brown, slightly paler medially. Legs: coxae reddish brown; trochanters reddish brown, paler; femora dark yellowish brown, with spines on fore and mid femora black; tibiae light yellowish brown; tarsi light yellowish brown, with basal and apical parts paler. Hemelytra light brown to dark brown; corium and clavus light brown, with cuneus slightly darker; membrane light greyish brown. Anterior margin of hind wings with dark brown shading; nervures dark brown (Fig. 2B).

#### Vestiture.

Body covered with golden hairs; upper surface, except shining head and anterior lobe of pronotum, matte; undersurface shining. Head covered with relatively sparse, uniformly long suberect setae; one pair of long setae arise behind each eye; antennae covered with long, suberect, pale hairs. Pronotum densely covered with adpressed to semi-erect short setae, with a single long seta at each anterolateral angle; mesoscutum and scutellum with dense, decumbent to suberect, short setae. Legs covered with long setae, relatively sparse on femora and dense on tibiae and tarsi; apex of front tibia with a tuft of hairs representing an incipient grasping organ and with a comb for cleaning the antennae (Fig. 3E). Corium and clavus with suberect setae, anterior and posterior margins of corium with long setae. Ventral side of the abdomen sparsely covered with setae.

#### Structure.

Head distinctly narrowed towards apex in dorsal view, slightly longer than its width; tylus broad, widening towards truncated apex, moderately prominent. Ocelli large, nearer to eyes than to each other. Antennae slender; segment I thicker and shorter than others; ratio of lengths of antennal segments I: II: III: IV = 1:1.94:1.54:1.89 (male) or 1:1.97:1.66:1.91 (female) (see Table 1). Labium slender, extending beyond fore coxae, with three visible segments; ratio of lengths of segments II: III: IV = 1:1.79:4.

**Table 1.** Measurements (in mm) of *Embiophilasinica* sp. nov.

Body part	Male (holotype)	Male (paratype, n = 2)	Female (paratypes, n = 3)
Length of body	1.94	1.94–1.96	2.17–2.25
Length of head	0.25	0.26	0.35–0.38
Width across eyes	0.30	0.29–0.30	0.34–0.35
Interocular space	0.14	0.13–0.15	0.20–0.22
Length of antennal segment I	0.11	0.12	0.14–0.15
Length of antennal segment II	0.23	0.24–0.25	0.24–0.26
Length of antennal segment III	0.21	0.21–0.23	0.20–0.23
Length of antennal segment IV	0.22	0.22–0.24	0.22–0.24
Length of labial segment II	0.09	0.07–0.08	0.13
Length of labial segment III	0.14	0.13–0.15	0.15–0.17
Length of labial segment IV	0.33	0.34–0.36	0.40–0.44
Maximum length of pronotum	0.49	0.51–0.53	0.53–0.56
Maximum width of pronotum	0.88	0.86–0.90	1.01–1.07
Length of fore femur	0.43	0.44–0.46	0.44–0.47
Length of fore tibia	0.40	0.39–0.41	0.40–0.43
Length of mid femur	0.45	0.44–0.47	0.47–0.52
Length of mid tibia	0.33	0.30–0.31	0.36–0.39
Length of hind femur	0.55	0.57–0.59	0.64–0.68
Length of hind tibia	0.61	0.62–0.63	0.65–0.68
Length of hemelytron	1.74	1.76–1.80	1.79–1.84
Length of abdomen	0.83	0.84–0.87	0.95–1.00
Maximum width of abdomen	0.67	0.69–0.71	0.70–0.74

Pronotum trapezoidal, with distinct collar, maximum width slightly less than twice maximum length; posterior lobe with lateral margins straight, posterior margin strongly excavated. Mesoscutum broadly exposed; scutellum wider than long at base. Legs relatively stout. Femora moderately thickened; fore and mid femora armed with small spines ventrally (Fig. 2C, D); hind femur with one row of bristles (Fig. 2E); fore femur armed ventrally with two rows of spines in apical third (Fig. 3C), each row with five spines; outside row with first spine long, second longer, apical three short and equal in length; inside row with first spine located between two larger spines at the base of outer row, second spine opposite to the second on outside row, remaining three short and small. Mid-femur armed ventrally with 11 spines in apical 4/5 (Fig. 3D), basal three spines extremely short, fourth to eighth stout, sixth to eighth slightly curved towards distal, and apical three short and adjacent, spines irregular at both base and apex; inside row with two spines short extremely, located between fifth and sixth on outside row. Tibiae straight; fore and mid tibiae widened towards apices; hind tibiae finely narrowed towards apex. Tarsi two-segmented (Fig. 3F); basal segment very short; apical segment long and slender, narrowed towards apex. Claws small, simple in shape.

Hemelytra broad, far exceeding apex of abdomen, with anterior margin expanding; costal fracture deep, located near apical three-fourths of corium (Fig. 2A); exocorium with about 60 corial glands (Fig. 3A, B); membrane broad, with chitinous thickening at base (not connected to cuneus), bearing single bristle basally. Venation of hind wings as shown in Fig. 2B; hind wings with a basal cell from which two nervures project: R + M not branched; free distal branch of nervure Cu, and Pcu free.

Abdomen oval, longer than its maximum width. Mediotergites membranous. Sterna fully sclerotized.

***Male genitalia*** (Fig. 2F–H): pygophore tubular, slightly curved, with basal part moderately rounded and bulging ventrally. Paramere symmetrical, long strip-shaped, slightly curved laterally, more nearly parallel-sided, about 10 times longer than its wide, apex with distinct constriction, distal tip curved laterally. Adeagus chitinized, acus elongate and slender, arcuate, gradually tapering to a fine point at apex with apex curved over. Anal segment semicircular.

***Female genitalia*** (Fig. 2I): female without ovipositor. First abdominal tergite with one pair of laterally opening copulatory tubes, unpigmented.

#### Etymology.

The specific epithet is a Latin adjective and refers to China, the country of the type locality of the new species.

#### Distribution.

**Figure 5.**
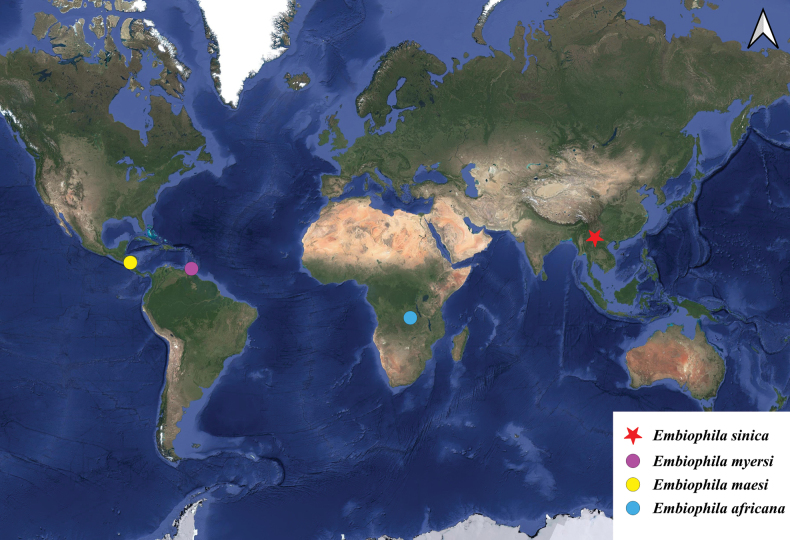
Known distribution of *Embiophila*: blue dot = *E.africana*; yellow dot = *E.maesi*; purple dot = *E.myersi*; red star = *E.sinica* sp. nov.

China (Yunnan: Pu’er) (Fig. 5).

#### Remarks.

According to Carayon (1974), this new species can be assigned to the subgenus Acladina (see Discussion for details).

### ﻿Key to species of *Embiophila*

**Table:** 

1	Labial segment IV more than twice as long as segment III; mid tibiae curved; hind wings with free distal branch of Cu; copulatory tubes absent in female	**2**
–	Labial segment IV less than or equal to twice of length of segment III; mid-tibiae straight; hind wings without free distal branch of Cu; copulatory tubes present in female	**3**
2	Fore femora armed with one row of seven spines; mid-femora armed with one row of eight spines; number of exocorial glands 35–40	***E.myersi***
–	Fore femora armed with two rows of spines (three spines per row); mid-femora armed with one row of seven spines; exocorial glands 13	***E.maesi***
3	Fore femora relatively slender, armed with two rows of spines, outer row with four and inner row with two; mid-femora armed with one row of nine spines; number of exocorial glands 10–20	***E.africana***
–	Fore femora relatively stout, armed with two rows of spines, outer row with 11 and inner row with two; mid femora armed with two rows of spines, outer row with 11 and inner row with two; exocorial glands more than 50	***E.sinica* sp. nov.**

## ﻿Discussion

In the seasonal rainforest in Pu’er, Yunnan, southwestern China, there are areas where tree leaves are relatively sparse, allowing sunlight to reach the moss and lichen-covered tree trunks. Embiopterans construct their nests there, with *Embiophilasinica* sp. nov. living on the web. Moss is not necessary, as we have also found traces of embiopterans and plokiophilid bugs on dry wood after logging. The number of adult bugs in each embiopteran web nest (approximately 1–7 adult embiopterans, and more than a dozen nymphs) is usually only 1–3, but nymphs can be quite high, reaching up to several dozen. The bugs exhibit strong locomotory capabilities, making them difficult to collect. When we strip away the webs for collecting, they quickly escape into the crevices of the trees. We collected some bugs from the field and fed them with mites (Acaridae) in the laboratory, and our observations once again confirmed the report by Carayon (1974) that the genus *Embiophila* feed on small arthropods within embiopteran webs. However, we did not observe them preying on embiopteran eggs, nymphs, and weak or dead adults, which still requires further observation. In the forest, with frequent rainfall, the webs of embiopterans provide safe and dry shelter for the web-lovers, while bugs help them clean various small arthropods, including mites, on the webs. Of course, these web-lovers can also survive independently of their hosts and can move freely on smooth surfaces without webs.

It is worth mentioning that from August 27 to September 12, 2024, we made two collections of *Embiophilasinica* sp. nov. at its type locality, collecting nine living female adults and numerous nymphs (at least 50), but no living male adults were found. The only male adult specimen was found to be dead on the web nest of embiopterans, and we speculated that it had been dead for a considerable amount of time. We believe that the males and females of the new species may exhibit significant differences in their emergence period and lifespan. Males in the same batch of nymphs are expected to emerge earlier than females, and they are likely to die shortly after mating, while females may survive for a longer period.

The known distribution of the four species of *Embiophila* is shown in Fig. 5. *Embiophilamyersi* and *E.maesi* are distributed in Latin America, while *E.africana* is found in Africa. *Embiophilasinica* sp. nov. represents the first record of *Embiophila* in Asia. According to Carayon (1974), *Embiophila* was further subdivided into two subgenera, *Acladina* and *Embiophila* s. str., based on the venation of the hind wings, presence or absence of copulatory tubes in females, the relative lengths of the labial segments, and the morphology of the mid tibiae. The subgenus Embiophila contains the New World species, while the subgenus Acladina is restricted to the Old World. The subgenus Acladina is characterized by the following diagnostic traits: labial segment IV less than or equal to twice the length of segment III; mid-tibiae straight; hind wings lacking free distal branch of Cu; and presence of copulatory tubes in females. In our study, *Embiophilasinica* sp. nov., collected from China, aligns morphologically with the subgenus Acladina, thereby corroborating Carayon’s classification. Additionally, Carayon (1974) noted that at least one undescribed species of *Acladina* exists in Laos, which requires further investigation.

*Embiophilasinica* sp. nov. is the third species of Plokiophilidae discovered in China, following *Plokiophiloidesbannaensis* Luo, Peng & Xie, 2021 and *Heissophilamacrotheleae* Schuh, 2006 (Yin et al. 2023). Near the collection site of *H.macrotheleae*, *P.bannaensis* was also found, and this location is only 80 km away from the area where *Embiophilasinica* sp. nov. was discovered. Although *Embiophila* and even the Plokiophilidae currently exhibit extremely limited species diversity, the clustered distribution of *E.sinica* sp. nov. with two known relatives (*H.macrotheleae* and *P.bannaensis*) within a geographic range of less than 100 km suggests that the tropical and subtropical regions in southern China and the adjacent Indochina Peninsula, characterized by similar habitat conditions (e.g., high temperature and humidity, highly diversity of symbiotic species), may harbor Plokiophilidae species diversity beyond current recognition. This group may have a pan-tropical distribution, although confirming this hypothesis requires intensified sampling efforts. Nevertheless, their dispersal mechanisms and evolutionary history warrant further investigation.

## Supplementary Material

XML Treatment for Embiophila

XML Treatment for Embiophila sinica

## ﻿Additional information

### Use of AI

No use of AI was reported.

### Author ORCIDs

Haoyang Xiong https://orcid.org/0000-0003-1086-5115

Zhuo Chen https://orcid.org/0000-0003-3393-0338

Hu Li https://orcid.org/0000-0001-8590-1753

Wanzhi Cai https://orcid.org/0000-0002-8620-0446

### Data availability

All of the data that support the findings of this study are available in the main text.
